# Book Reviews

**Published:** 1829-01

**Authors:** 


### CRITICAL ANALYSES.

Quæ laudanda forent, et quæ culpanda, vicissim
Illa, prius, cretâ; mox hæc, carbone, *notamus.*—PERSIUS.

*A Manual on Midwifery; or, a Summary of the Science and Art of Obstetric Medicine; including the Anatomy, Physiology, Pathology, and Therapeutics, peculiar to Females; Treatment of Parturition, Puerperal and Infantile Diseases; and an Exposition of Obstetrico-legal Medicine.* By MICHAEL RYAN, M.D. &c. &c. &c.—8vo. pp. 353. London: Longman and Co. 1828.

*A Practical Treatise on Parturition, comprising the attendant Circumstances and Diseases of the Pregnant and Puerperal States.* By SAMUEL ASHWELL, Member of the Royal College of Surgeons, and of the Medico-Chirurgical Society of London. *To which are appended two Papers, the one containing some Remarks on Abdominal Surgery, the other on Transfusion; presented by* Dr. BLUNDELL, of Guy's Hospital.—8vo. pp. 546. London: T. Tegg, 1828.

THE tone of self-complacency which runs through the preface of the first of these volumes is highly amusing. It is quite impossible that the public can estimate the value of the work at a higher rate than the author does; and if they only keep pace with him in the eulogiums he has so liberally bestowed upon himself, he cannot with reason be dissatisfied. Dr. RYAN's opinion of Dr. Ryan's book is, that "there is no other work of this size, foreign or national, that contains so much practical information." The classification, also, "is more scientific than that of any other writer." Notwithstanding the encomiums he has lavished upon himself, the author trusts that he "will not be deemed egotistical!" Upon this point, however, the verdict must be against him, or we misapprehend the meaning of the term.

Dr. R. piques himself for having treated very fully the disputed question of the power of the mother's imagination on the infant; and he thinks he has exposed the ridiculous stories of ruder ages, and succeeded in establishing the *negative* opinion on the subject. Will he forgive us if, without "a few words apologetic," (to use his own phrase,) we suggest to him that, in future, he need not labour to prove the negative of a proposition, unless he can find somebody to support the affirmative. Errors which have been long abandoned demand no longer formal exposure.

"Official duties" at a crowded dispensary, and "innumerable interruptions attendant on private practice," have prevented the author from paying as much attention to the style and arrangement of the work as he could have wished: he therefore thinks "allowances" ought to be made. But there has been no compulsion in the case: Dr. Ryan has not been *obliged* to write under so extraordinary a pressure of occupation, and cannot, therefore, fairly avail himself of the plea of such interruptions as an excuse for the imperfections his work contains.

As a book of reference for beginners, for which such a work must be principally intended, Dr. Ryan's volume is eminently defective. The arrangement of most of the subjects is faulty and confused: various points of discussion, which have no connexion with each other, follow under the same head in so heterogeneous a manner, that the student must expend much time before he could find the subject he was in search of. The author, indeed, presumes that the work affords great advantages to the student; "for he may fairly consider it a review of the ancient and most modern works on obstetric medicine." Now we apprehend that a "pocket companion" for such a class of readers should give a clear and practical sketch of the subjects it contains, and that it is injudicious and useless to encumber it by enumerating a great variety of discrepant opinions and hypotheses, which may be interesting for the senior practitioner to look over, but which will certainly tend to damp the ardor of a beginner, and to confuse his mind. Neither can we commend the introduction of a number of new and unnecessary terms, such as "gynæcophysiology," "parthenosology," "lochianosology," &c. We are not in the habit of examining with much critical severity the style in which a medical writer delivers his sentiments. It would not be difficult to enumerate many authors who have made very important additions to our stock of information in very unpolished language; but, in more than one division of Dr. Ryan's work, we observe, with much regret, that he indulges in a wantonness of phrase which, as a lecturer, he ought studiously to have avoided. He informs us that the ancients maintained that "the mind should be sober and chaste" while acquiring a knowledge of the structure of females. In his chapter upon "gynæcophysiology!" our modern author, however, has not evinced either much sobriety of expression or chastity of thought.

From the numerous references Dr. Ryan makes to a long list of authorities, it is very evident that he has given much time to the study of his subject, with which he may be generally well acquainted. Every page abounds with quotations from various sources, but unfortunately they are so loosely and carelessly thrown together, that the negligence of the author as to method destroys the advantages which might have resulted from his industrious researches. *Undique collatis membris* is stamped in every part of the book, but the members are too unskilfully united to form either an agreeable or instructive whole.

The design of Mr. Ashwell has been to steer a middle course between the large and comprehensive systems of Baudelocque, Denman, and Burns, and the mere outlines and manuals upon the obstetric art. Drs. Blundell and Marshall Hall have afforded the author many valuable suggestions and most important facts. That part of the work which treats of puerperal diseases has been carefully examined by the latter. Dr. E. J. Hopkins and Dr. F. Ramsbotham have also contributed their assistance.

"The history of midwifery," with which the volume commences, might have been spared: it has, however, the merit of brevity.

Part I. "The Obstetric Properties of the Pelvis, carefully noticing those deviations which may obstruct Parturition." The anatomy of the pelvis is considered only as far as it is essentially connected with parturition and its consequences.

Much difference of opinion has existed as to the separation of the bones of the pelvis previous to or during labour. It is said to be a fact that, in many mammiferous animals, immediately previous to labour, a relaxation of the ligaments, producing a separation of the joints, does occur. Ruysch and Harvey, judging from their own observations, were convinced that a similar separation almost invariably takes place in the human female. Denman was inclined to believe that the degrees of separation of the junctions of the bones of the pelvis may be very different, and that when it proceeds beyond a certain degree it is to be considered as morbid. Smellie asserts that this separation is very uncommon. Mr. Ashwell is not inclined to believe that any such invariable relaxation of the joints of the pelvis, as would materially facilitate the progress of difficult labour, forms any part of the parturient process. Such is the general opinion, and our own experience convinces us that it is correct.

The following observations should not be forgotten by the junior practitioner. In more than one instance we have known very erroneous opinions given as to the probable duration of labour, from want of reflection upon so obvious a fact.

"The depth of the pelvis is a point of consequence, more especially in estimating the progress of labour. We find at the symphysis pubis it does not exceed an inch and a half, or at most two inches, posteriorly from the base of the sacrum to the point of the os coccygis; when under pressure, it is about four times as deep as in front; and laterally the depth of the inferior extremities of the ischia is about four inches. Bearing these facts in mind, we shall not always allow ourselves to imagine, because we can readily feel the child's head in the front of the pelvis, that its birth is instantly to take place; but, remembering the intermediate depth of the pelvis laterally, and its very much greater depth posteriorly, we shall be fully aware that difficulty may be experienced from incarceration. In making examinations, it is well to avail ourselves of the shallowness of the pelvis in front, which affords the utmost facility for carrying the fingers considerably beyond the brim." (P. 51.)

We pass over much elementary matter upon the subject of deformity of the pelvis, of pelvimeters, and of the structure and dimensions of the head of the child. It is scarcely necessary we should observe, that they demand the careful consideration of the student.

Part II. comprises "Menstruation, the description of the Gravid Uterus, with the doctrines of Conception, Sterility, and the Signs and Diseases of Pregnancy."

The nature of the menstrual fluid is briefly considered. As a proof that it does not coagulate, it is observed, that Dr. Mansfield Clarke, in his lecture on this subject, exhibited a specimen of the menstrual discharge which had remained in a fluid state for many years. Mr. Ashwell states, "that the catamenia are invariably suppressed in pregnancy." That such is the fact in a very great majority of cases, is, we believe, universally admitted: that occasional exceptions happen to this, as to every other general rule, we are, however, fully convinced from our own personal experience. This subject is by no means unimportant; for, if the practitioner *positively* determines that a woman is *not* pregnant because she menstruates regularly, he will sooner or later discover his error. It appears that Mr. A. has but carelessly referred to the observations of Dr. Dewees, who not only "adduces cases in which the catamenial discharge did regularly appear during the first three or four months of pregnancy," but who also states that "we are perfectly familiar with a number of women who habitually menstruate during pregnancy, until a certain period; but when that time arrives, it ceases: several of these menstruated until the second or third month; others longer; *and two until the seventh month.* The two last were mother and daughter."* Dr. Dewees very strictly questioned these patients, who could have no motive for deceiving him, and he declares his conviction that there could be no mistake.

In support of the opinion that women do sometimes menstruate during pregnancy, we give the following passage from Dr. Ryan's work:

"The menses may appear regularly in the first seven months of gestation, according to Dewees, who contends that Hunter, Baudelocque, and Burns, are of opinion that the uterus is not closed by decidua during the first two months of pregnancy. The neck of the womb is free, and hence menstruation may happen; and Dr. Coxe had a patient who menstruated regularly, and in whom there was not more than the size of a thumb-nail of healthy surface in the womb. Dr. Dewees knew a number of women who menstruated during the early months, and a mother and daughter who continued to do so to the seventh month. Dr. Heberden knew a lady who menstruated regularly during four pregnancies. Haller, Hosack, and Francis were of the same opinion. Dr. Dewees knew a woman who menstruated only during pregnancy; also Deventer, Fodere, and Capuron. Dr. Hamilton denies the possibility of the occurrence."

We are only desirous of declaring the occasional continuance of menstruation during pregnancy. We perfectly agree with Mr. Ashwell, that, "for ourselves, we should feel more confident of the existence of pregnancy from an entire suppression of the catamenia, all other signs, with the exception of the abdominal increase, being absent, than we should from the united assemblage of all the other indications, if the catamenial secretion continued with its accustomed regularity, and of its natural character."

* Dewees' Midwifery, p. 96.

We pass over several chapters, which contain much elementary information, conveyed in a concise, yet perspicuous and practical form.

The author observes, that "a diversity of practice has obtained in the management of the membranes, some practitioners invariably leaving their rupture to the natural efforts, while others as invariably break them by artificial means, so soon as they are within reach, and before the dilatation of the os uteri is fully accomplished." What "some practitioners" may do, we cannot venture to determine; but we know no respectable authority who lays down either of these practical rules. To rupture the membranes as soon as they are within reach, must frequently be decidedly improper. But when the pains are strong, and the os uteri well dilated, or that it is judged, from the feel of it, that it is easily dilatable, they may then be ruptured without any hazard of protracting the labour: on the contrary, under these circumstances the parturient process is often more speedily terminated.

Upon this subject Mr. Ashwell observes:

"The rule should be, to leave their rupture to the natural efforts; the exception, to produce it by artificial means. There are two instances in which the rupture of the cyst is fully justifiable: first, when, at the sixth or seventh month, there is an attempt to throw off the ovum entire and unbroken, in which case there might be most alarming hemorrhage from the placental vessels, and the fœtus might probably be drowned in its own waters. And, again, when the membranes are unusually tough and unyielding. Here we have known labour delayed several hours from an unwillingness to interfere with its natural progress. If, however, we find the membranes pushed down along the vagina, and protruding beyond the vulva, we may feel assured the os uteri is fully expanded; and we cannot err if, during the height of a pain, we force a stilette through the cyst, after which the child will sometimes be almost immediately born." (P. 250.)

The use of any instrument unnecessarily should at all times be avoided, and, as the membranes may just as easily be ruptured by the finger, or scratched through with the nail, we should not think of using a stilette.

We regret to find it stated in a work designed for the guidance of the student, that "in labours generally it is of very little importance whether we know the presentation or not, as it is most commonly a natural one, and the birth will safely occur independently of our assistance." It is very true that the author follows up this observation, which had been much better omitted, by pointing out the necessity of promptly ascertaining the presentation. Still the previous remark holds out an excuse for carelessness and inattention, which would have been wisely avoided.

From the following passage it would be inferred that the practitioner is not called upon to ascertain the presentation until *after* the rupture of the membranes. "Supposing the membranes to have been ruptured, and the liquor amnii to have escaped, the first stage of parturition is completed, and the very important duty of ascertaining the presentation devolves upon the accoucheur." (P. 250.) It is very evident that the author is sensible of the importance of determining the presentation *before* the rupture of the membranes; for he has previously laid it down as a rule in midwifery, to see the patient about to be confined as early as possible, "for there may be a preternatural presentation, and, from the rupture of the membranes and the escape of the water, the favorable moment for turning may be lost previously to the arrival of the accoucheur." We have read and reread the sentence above quoted, and can attach to it no other meaning than that we have assigned, although it is clear Mr. Ashwell does not mean what he says He is aware that the important duty to which he refers devolves upon the accoucheur, not when the liquor amnii has escaped, but *before* it has escaped, although the above sentence, taken alone, expresses the contrary opinion.

The author observes, that "some practitioners are very particular in their directions about the membranes, and we invariably attempt, by a very gentle withdrawal of the placenta, to secure the complete extraction of the secundines; thus obviating the subsequently painful contractile efforts to expel them, and at the same time removing a source of annoyance, in the offensive smell, to which, by their putridity, they may give rise."

The complete removal of the secundines, which is of much consequence, from the circumstances Mr. A. mentions, is rendered more certain by turning the placenta round once or twice in the act of extracting it. By this simple expedient the membranes are twisted, and are therefore less likely to break from the placenta, and to leave some portions still remaining in the uterus.

Mr. Ashwell adds his testimony to the almost invariable power of the ergot of rye in increasing uterine action. He does not promise infallible success from the use of it. He gives a brief and perspicuous summary of what is known of the powers of this remedy.*

* Mr. MICHELL's work on Difficult Labour, reviewed in our Number for August 1828, contains the most satisfactory body of evidence upon the use and efficacy of the ergot of rye.—REV.

Mr. Ashwell offers some judicious remarks upon tedious labour from rigidity of the soft parts. Unless in extreme cases, he would not abstract blood so largely as Mauriceau and Dewees have recommended.

"Were we called to a patient with rigidity, whose previous labours had been protracted from the same cause, we should consider venesection as the most important in the series of remedies. In rigidity, however, of a moderate kind, we should first empty the rectum, whose feculent accumulations frequently obstruct labour. The bladder should not be distended, and the erect posture, short of fatigue, may be maintained; every thing being avoided at all likely to produce fever. Tea, toast-and-water, barley-water, milk-and-water, or veal-broth, may be taken; and the apartment should be airy and cool. We have twice rubbed in the belladonna, as advised by Dr. Conquest, but without any benefit. We have many times been much gratified with the effect of opiate clysters, or suppositories, introduced into the rectum, even after opium had been taken internally without any apparently good effect. Opium should not be given until the bowels have been relieved; and, of course, if bleeding has been previously employed, it will be exhibited under the most advantageous circumstances." (P. 273.)

We by no means presume, with the author, that the long forceps will ultimately entirely supersede the short forceps.

In the following general observations upon instrumental assistance, we fully concur. We have seen many patients who have been allowed to suffer for hours, when the labour might have been terminated in a few minutes by the use of the forceps, with perfect safety both to the mother and child, with due dexterity and experience on the part of the practitioner.

"All instruments may be rendered dangerous, if too early and rashly used; yet we think that experience is decidedly in favor of the greater safety to the mother from their too early, than from their procrastinated employment. Rupture of the uterus, abdominal and local inflammation, terminating in gangrene and sloughing, irreparable exhaustion of the system, and a series of other events not necessary to be enumerated here, may all be occasioned by a too protracted difficult labour. Indeed, we are sometimes almost induced to believe that great evil has arisen from the multiplied and fearful associations which have been so invariably connected with the use of instruments. Some practitioners are thereby deterred from even thinking of their employment, till a period has approached when little good can be anticipated from their aid. Others consider it so superlatively difficult to determine the cases proper for their use, and the precise time and manner of their application, that they think it unnecessary to acquire a thorough knowledge of the principles on which instrumental labour can alone successfully proceed, not remembering that, in some instances, valuable lives may be entirely dependent on their sole and unaided exertions, and that, before they can obtain the assistance of another practitioner, their secret source of reliance, the proper moment for interference may have been finally lost. In the difficult operations of surgery, those of hernia and lithotomy, by way of example, the circumstances in which they are to be performed are fully stated, the dangerous occurrences attending their execution, and the methods of averting or of contending against them, if they do happen, are carefully described, and, having acquired a knowledge of every possible contingency, the operator is well prepared to meet every difficulty. Nothing beyond this is required in instrumental parturition. Let it be understood, that although very rarely, yet that sometimes artificial aid is necessary; that it behoves the accoucheur accurately to discover the nature of the difficulty opposing delivery, and how far it is likely to be overcome by the natural efforts; that, if he deliberately determines these to be insufficient, he is next to ascertain the precise situation of the child's head in reference to the pelvis; and if the os uteri be fully dilated, he may proceed by the forceps, as by a pair of artificial hands, to obtain a firm hold of the cranium. That, in their introduction, he is to be guided by certain directions clearly and simply taught; and, in the subsequent extraction, he is to act on principles arising out of the relations of the bony canal of the pelvis to the head of the child. That, in the performance of these duties, he may encounter greater or less difficulty, and in some instances the obstacles may prove insuperable; yet, if gentleness and caution be observed, and a strict regard paid to the axis of that part of the pelvis in which the difficulty exists, he may advance from the moderate to the higher degrees of contracting power, without any injury either to the mother or her offspring."

In offering our approval of the sentiments here expressed, we are very far from recommending a hasty recourse to the use of instruments: but to forbear from employing them when they are necessary, is quite as injudicious and discreditable to the practitioner as to use them when they are not.

Although every opinion stated by Dr. Gooch deserves our serious consideration, we agree with Mr. Ashwell that the practice he recommends in flooding after the removal of the placenta ought not to be adopted as a general rule. Dr. Gooch states, "that, when hemorrhage occurs after the removal of the placenta, the quickest way to stop it is to introduce the left hand closed within the uterus, applying the right hand open to the outside of the abdomen, and then, between the two, to compress the part where the placenta was attached, and from which chiefly the blood is flowing." In answer to this, Mr. Ashwell prudently replies—

"I am aware that, in alarming and desperate floodings, any measure, however severe, is justifiable: the intention is to save a life which appears on the very point of extinction, and after consequences must yield to this momentous purpose. I do not deny that, as a 'denier resort,' the carrying of the hand into the uterus may be absolutely necessary; but I am equally convinced that the griping or grasping pressure of the womb, commenced immediately after the birth of the placenta, when there is an habitual proneness to flooding, or when the contraction of the uterus is unsatisfactory, will generally supersede its employment. The introduction of the hand into the uterine cavity is always attended with risk, and it cannot be less so when, owing to the exhausted and powerless state of the system, the uterus and vagina may easily suffer rupture or laceration. It may be, too, urged against this practice, when it is performed during syncope or approaching collapse, that it must of necessity destroy coagula or clots, which are forming about the mouths of the bleeding vessels, and may thus originate fresh hemorrhage." (P. 448.)

Ample experience teaches us that Mr. A. has very judiciously qualified the rule of practice laid down by Dr. Gooch.

It behoves the student and junior practitioner to consider most maturely the various circumstances connected with the subject of uterine hemorrhage, that he may not have reason to regret his want of information at a moment when the life of his patient may be entirely dependent upon his presence of mind and prompt assistance.

In the fourth part are considered "the Diseases which belong to the Puerperal State." The following passage is worthy of attention:

"On visiting a patient a few hours after delivery, we shall almost invariably observe a slight but complete febrile paroxysm, characterised by quickness of pulse and general heat of surface; and, if stimulants be not administered, perspiration usually ensues, and in twenty-four, forty-eight, or seventy-two hours, the affection wholly subsides. I have known this condition produce unnecessary alarm, and I am convinced it has often, to the injury of the patient, prompted to the abstraction of blood and other unnecessary depletory measures. It ought, when moderate, to be regarded as a natural effect of the shock incident to parturition." (P. 453.)

The morbid occurrences of the puerperal state are but briefly touched upon. Upon this subject Mr. Ashwell has freely quoted from Dr. Marshall Hall's excellent publication on "some Diseases incident to the Puerperal State."

In an Appendix two papers are contained, relative to the "Surgery of the Abdomen," and on "Transfusion." For these communications Mr. Ashwell is indebted to Dr.

Blundell. The plates in this work are neatly executed, and will be found very useful to the student, in impressing upon his mind a clearer notion of the various positions of the fœtus in utero than any verbal description could convey. They are principally, if not entirely, copied, upon a diminished scale, from the large work of Smellie. In the language of the printing office, the work is well "got up."

The author has achieved the object he had in view, of giving a brief summary of the various subjects upon which he treats. As a *preliminary* study, his work may be consulted with some advantage by those who fear to enter at once upon the perusal of other authors, who treat the same subject in a more comprehensive manner.

---

*Commentaries on the Causes, Forms, Symptoms, and Treatment, Moral and Medical, of Insanity.* By George Man Burrows, M.D. Member of the Royal College of Physicians of London, &c.—8vo. pp. 716. Thos. and Geo. Underwood, London, 1828.

(Concluded from page 544 of the last volume.)

Every page of Dr. Burrows' work contains discussions of so much practical importance, that we very unwillingly pass over any part of it. But it would be impossible, within the ordinary limits of our analyses, to notice each of his very interesting commentaries. We have already exceeded our usual bounds, but still we have but skimmed along the surface. The volume itself must be perused, and maturely studied. It will be found not only to impart information upon the subject to which it is particularly devoted, but, from the reciprocal connexion between the brain and all other parts of the body, many general pathological views are incidentally noticed, which bear upon various other maladies as well as insanity.

Commentary III. "Delirium."—The author gives a summary of the opinions of various writers, who have endeavoured to draw a line between delirium and insanity. This distinction is very important, for, as different affections derived from different states of the brain, they require opposite modes of treatment.

Commentary IV. contains a succinct practical sketch of "Delirium Tremens." Dr. Burrows has met with several fatal cases. "In three of them which were examined, each dissection displayed considerable venous congestion and effusion of serum. In one man, who died very suddenly, between the membranes, in the ventricles, and the theca vertebralis, there was an immense accumulation of serum."

In respect to treatment, the author remarks "I have treated such patients by opiates, and without any narcotic at all; and they have by both modes recovered in the time this disease usually occupies. Formerly, if the constitution were not broken down by a long-continued habit of intoxication, but on the contrary appeared rather full, I prescribed bloodletting from the arm; but never finding any corresponding benefit, I long since ceased the practice. Afterwards I tried abstracting smaller quantities of blood from the occiput or nape of the neck, by cupping; or from the temples, or behind the ears, by leeches, in order to reduce the cerebral action; and applied an evaporating lotion to the head. Moderate depletion and cooling applications will generally relieve and refresh a vigorous young patient; but must be cautiously adopted if an old one. As the bowels are often constipated, and the secretions bad, moderate purging is almost always indicated." (P. 332.)

The quantity of opium to be given must be regulated by circumstances. Dr. B. generally prescribes three grains in the first instance, and continues it in smaller doses every hour or two till sleep is obtained. If the disease is induced by a total deprivation of an accustomed stimulus, a little should be given now and then. Camphor is frequently useful as an excitant.

Commentary V. treats of the "Stages of Insanity." Other diseases which are properly called acute, if not interrupted, have their incipient stage, and, in succession, those of intensity and convalescence. Such diseases, also, may assume a continued, remittent, or intermittent form. "So likewise may insanity. Thus far, then, the analogy of insanity and acute diseases holds, but no further; for, though the former be an active disease, inasmuch as it runs through distinct stages, it cannot, in the sense in which acute is applied to other diseases, pretend to that character." (P. 340.)

For the philosophic division of insanity, as a corporeal disease, into stages or periods, we are indebted to the observation and discrimination of the celebrated Pinel, and it has been recognised by his successors. Dr. Burrows is convinced that the neglect or oversight of the different stages or periods of insanity, is the principal cause of the confusion, vacillation, and frequent disappointment in the remedial treatment of it. He therefore follows the example of the French pathologists, and describes the successive periods into which the disorder divides itself, and should be studied. It is frequently to be lamented that the physician is not consulted until it is too late to prevent the full development of the malady.

Commentary VI. "Puerperal Insanity."—Upon this very interesting form of mental derangement, Dr. Burrows deduces from his experience, which has evidently been considerable, the following corollaries:

"1. That mania is a more frequent consequence of lying-in, and the process of lactation, than any other variety of mental derangement.

"2. That puerperal insanity occurs from the age of twenty to thirty, in the proportion nearly of two to one at all other ages.

"3. That, in London, physical causes much more frequently originate puerperal insanity than moral causes; the physical being to the moral as ten to one. In Paris the reverse obtains, and the moral are to the physical as four to one.

"4. That the access of puerperal insanity happens before the fourteenth day in three out of five cases.

"5. That it happens between the fourteenth and twenty-eighth days in one out of about six cases and a half.

"6. That nearly four in five recover their intellects.

"7. That not more than half recover in six months.

"8. That those recover soonest whose delirium supervenes on the process of lactation.

"9. That the maniacal form ceases sooner than the melancholic.

"10. That the mortality is apparently, but not really (as will be proved presently,) double Esquirol's return; and that the greater number of deaths occurred before the second week from delivery.

"11. That half (and possibly more, if the truth could always be discovered,) attacked by puerperal insanity, prove to possess an hereditary predisposition." (P. 396.)

Dr. Burrows attaches but little weight to the cases of mortality reported by Esquirol. Not one of them occurred till more than six months from the access of the insanity, and in others, years had intervened. Puerperal insanity is considered to be a dangerous disorder, on which a very cautious prognosis should be delivered.

*Treatment.*—The actual situation of the patient must be kept in mind. Denman sensibly observes, that when a woman is recently delivered, the attending circumstances reduce her to the state of a person who has had a profuse evacuation of any other kind. Such patients may require depletion in different acute disorders, but they generally do not bear it well. Care must be taken not to mistake symptoms of excitation for inflammation, or muscular exertion for vital power.

"When called to a case of this nature which has occurred within the month following lying-in, I cannot too forcibly impress the remembrance that the puerperal patient is already reduced by parturition and its consequences; and that the process of lactation itself produces fever and considerable irritation, both of which will ordinarily subside in a few days, if the bowels be opened, and the milk have a natural vent, or be duly carried off, when, from accident, suckling is impracticable." (P. 399.)

Influenced by these views, the author, of course, does not consider that depletion, or the reduction of strength, is the proper course to restore the equilibrium of those functions on which health and a sane mind depend.

"With pain I must acknowledge that I have too often found, when called to a case of puerperal insanity, that the sins of commission in the treatment of it have been infinitely greater than those of omission; for in most of them depletory measures have been pushed to an unreasonable extent, so that the issue was already perhaps determined before I was consulted, and no alternative left but death or long-continued insanity. And to this cause, I fear, must be ascribed a larger proportion of mortality consequent on puerperal insanity, than would result if a more cautious system of practice were adopted." (P. 399.)

Puerperal mania is exhibited in two forms, each distinct in their physical characters. In one the delirium is high, with ordinary excitation; in the other the delirium is low, with symptoms of cerebral disease, with coma.

"The first, if properly treated, is attended perhaps by little danger either of life or continued insanity: the second is attended by great danger under any treatment; and, if life be saved, it is commonly at the expense of reason." (P. 401.)

If the secretion of milk and the lochia are suspended, it will be desirable to restore them, if possible. Dr. B. has seen suppuration of the breasts prove critical, and many other abscesses.

When the insanity is fully developed, the first duty is to prevent the patient from injuring herself or others. The bowels must next be freely evacuated. If purging weakens, clysters must be employed. If the delirium is of a more determinate character, local bleeding on the occiput, vertex, temples, or behind the ears, and cold evaporating lotions to the head, will be necessary.

"The pulse, as well as the muscular movements, in this and in all other species of mental affection, as I have before remarked, is commonly referred to as the index of the strength of the patient. They are both equally fallacious signs, and must never be trusted in these more than in any other cases of insanity." (P. 403.)

The lower extremities must be kept warm. A foot bath, filled with a warm infusion of mustard seed or of horse radish, may be used with advantage. The use of opiates and blisters requires much discrimination. Determination or congestion of the cerebral vessels, and a costive state of the bowels, must first be remedied before opiates will produce the desired effect, and even then cold applications to the shaven head will be the best and most certain soporific. Having attended to these preliminary measures, if the cold applications fail to produce the effect, opiates must be freely administered. Small doses only increase the irritation and delirium. Battley's liquor opii sedativus is preferred by the author. Upon more than one occasion we have expressed our favorable opinion of this form of opiate, particularly in cases where cerebral excitement was to be guarded against as much as possible. Blisters, either to or near the head, are not advised. "The only way in which I have thought advantage has been produced by them is as a derivant, when applied to the thighs or legs." Some physicians ridicule the theory upon which this derivative treatment is founded, and doubt the efficacy of the practice which results from it. From frequent observation, however, we are convinced that blisters thus applied are serviceable in such cases, but prejudicial if applied to the head.

Care must be taken that some nutriment is got down, "for a sudden and most unexpected state of exhaustion frequently supervenes, and may carry off the patient."

The moral treatment must be conducted upon the same principles as in general insanity.

In the seventh Commentary, the author touches briefly upon that species of mental aberration peculiar to old age, and hence designated *delirium senile*, or *senile insanity*.

"The whole moral and intellectual character of the patient is changed: the pious become impious, the content and happy discontent and miserable, the prudent and economical imprudent and ridiculously profuse, the liberal penurious, the sober drunken, &c. Persons in whom the sexual passion has been long dormant suddenly become lascivious and obscene, and abandon themselves to all sorts of vices. In fact, the reverence which age, and the conduct suited to it, always commands, is converted into shame and pity at the perversion of those moral and social qualities which, perhaps, have hitherto adorned the decline of the patient's days, and endeared him to his family and friends." (P. 409.

This form of disease develops itself in those who may never before have been insane, nor possess hereditary predisposition. The treatment must generally be purely palliative.

Commentary VIII. "Suicide."—Many contend that the remote causes of suicide exist always in some lesion or disease of the thoracic or abdominal viscera. Dr. Burrows does not dispute that this may sometimes be the fact, because he believes that it is so frequently where there is general insanity, without propensity to suicide. "But certainly neither in the encephalon nor in other viscera has any lesion or disease been detected, which peculiarly characterises suicide." Falret* infers that the affections of the viscera, in mental derangement, are always secondary, while the primary affection is in the encephalon. To this the author replies, very fairly, that

"If the viscera are affected secondarily from a morbid action or disease in the brain, I know not by what reasoning we should deny a reciprocal influence, and that the brain also may be secondarily affected. Although actual disease of the liver in cases of suicide be rare, and concretions are seldom found, yet a diseased hepatic action may exist, and the ducts in consequence be irritated by the passage of vitiated bile; and hence the brain, through the nervous influence, be sympathetically affected, and the mental depression prompting suicide induced." (P. 415.)

The strictest anatomical researches have elicited no other evidence than what corresponds with the general pathology of mental derangement.†

"Sometimes the patient makes no secret of this unhappy propensity. At this time a lady is under my care in whom insanity is hereditary. Her case is mania, alternating with melancholia, and when in the latter state the suicidical disposition comes on. She is perfectly conscious of her condition, reasons upon and laments her extravagant actions or gloomy ideas, and piteously begs she may not be trusted." (P. 421.)

It must be carefully remembered that these reasoning lunatics are not to be trusted. As a proof of the deliberate determination with which the means of self-destruction are obtained, the following facts are mentioned:

"A woman, named Wild, occupied several weeks in purchasing such small quantities of oxymuriate of mercury as to avoid the suspicion of her purpose. She then administered enough to her three children and herself as to cause the death of all of them.

"A gentleman obtained daily one grain of opium for eighty days, under pretence that he could not sleep without it. He then swallowed the whole with the intent of destroying himself."

*Treatment of suicide.*

"The medical treatment of the propensity to suicide, whether prophylactic or therapeutic, differs not from that which is applicable in cases of ordinary insanity. If suicide be the accompaniment of mania, or of melancholia, the remedies must be such as are suitable to those states of mental disorder, without reference to this specific symptom. The only difference is this, that in cases of insanity, when marked with violence, the precautionary means are to prevent mischief to others, and, when marked by disposition to

* Essai sur le Suicide. Paris, 1822.

† Vide page 529 of our last Number, for extracts from Dr. Burrows' work upon this subject.

suicide, to protect the patient himself; and that, in the latter case, a much greater degree of vigilance is necessary." (P. 449.)

Melancholy patients, it is said, have had the morbid association of their ideas broken, or long-continued hallucination chased away, by exciting some sudden and violent emotion. Dr. Burrows has never ventured to try the effect of surprises or fright. Before such experiments are practised on the insane, he cautions us "attentively to consider the state of the patient, especially that no cerebral congestion exists, lest apoplexy should close the scene."

The difficulty of determining when we may with safety place confidence in a convalescent suicide, must be obvious. Dr. F. Willis was once called upon for the utmost presence of mind.

"The late king desired one day to shave himself. Willis feared that, if he hesitated to give his consent, the king would see that he was suspected of an intention to commit suicide, and thus the idea of such an act would be engendered where it might not as yet exist. He promptly sent for the razors; but, before they could be brought, he engaged his majesty's attention with papers which were upon the table. The king continued so occupied with them, that his physician felt assured he entertained no design of the kind. After having shaved himself, he resumed his papers. The razors were not sent away immediately, lest the thought should come across the king that he could not be trusted. Such self-possession and tact would have been admirable in an ordinary case; but when we consider the rank of the patient, and the immense responsibility attached, we must own that Willis was endowed with exemplary qualifications for the trust imposed upon him." (P. 461.)

We recommend the whole of the commentary upon the dreadful tendency to suicide particularly to the attention of our readers.

We must pass on to Part V. which discusses the "curative treatment." The author professes no knowledge of an antimaniacal remedy, nor does he offer the charm of novelty in his "plans or practices." His practice has been directed by those pathological views of the causes, nature, forms, and complications of insanity, which he has so extensively described. He conceives that every case of insanity should be considered as an insulated one, and so it must be treated. "Remedies, therefore, must vary with the constitution and peculiar features of each case." No fixed rules or formulæ can be given, or, if prescribed, be adhered to. Without any wish to defraud the moderns of what is owing to them in developing the causes, or improving the treatment, of insanity, the author considers it due to the ancients to acknowledge that their practice was generally judicious, and that he feels more indebted to them than to the moderns for the success which has attended his efforts.

Like the causes of insanity, the curative treatment is usually divided into medical and moral; and first of the "*medical treatment.*" The first duty of the physician will be to make himself acquainted with the history of the patient and the case. Success in the treatment of insanity, as in other diseases, is always correspondent with the interval between the attack and the period when remedial care commences. It must be remembered that insanity is a purely corporeal disease, and, like other corporeal diseases, is amenable to medical skill.

"As the functions of the vascular or nervous system, in all cases of insanity, are disturbed, our skill should be directed, while a prospect of cure is entertained, to diminish the action of either system which may preponderate, so as to restore the lost balance.

"When the cause evidently exists in a structural or functional lesion of some remote organ, affecting the brain by sympathy, it is obvious that attention must be first directed to the organ so affected." (P. 578.)

The several stages which insanity pursues in its course testify that the brain, the organ of the mind, assumes different morbid conditions, first functional, and then structural; functional in the first three stages, structural or organic in the last.

"This pathological view must be our guide in prescribing.

"In the incipient stage, there is evidence of great vascular excitation and cerebral irritation; and this stage must be met by a correspondent treatment. Here are indicated repeated topical abstractions of blood from the head or contiguous to it, shaving the head and refrigeration so long as there is preternatural heat of the scalp; cautious general bloodletting, even in the plethoric and robust, very moderate in the delicate though young; purging; vomiting after the vessels of the head are unloaded and the bowels evacuated; nauseating doses of tartarised antimony, to moderate the circulation and excessive violence; the digitalis in gradually augmenting doses, till the pulse intimates reducing the dose; saline draughts, and moderate diet.

In the active or confirmed stage, the fury and violence of mania, or the despair of melancholia, with their concomitant mental delusions, may persist, yet the symptoms of physical excitation attending the incipient stage subside or intermit, and occasionally only return.

"When the symptoms of excitation recur, they must be treated as in the first instance, except that neither depletion by local or general bleeding, nor by any evacuants, should be so active or copious. The system will not in this stage bear them so well; on the contrary, light tonics and the shower-bath are of great use, even when moderate topical bleeding and purging are indicated; and, when the exacerbation of a paroxysm ceases, more powerful tonics, as chalybeates, cinchona, cold bathing, and a better diet, are admissible. It should also be observed, that in melancholia the class of remedies which are designated anti-nervines are useful adjuvants.

"In the convalescent stage, if symptoms still denote cerebral congestion, gastric irritation or uneasiness, or intestinal irregularities, they should be attended to until they are removed. In this stage, moral treatment besides is especially indicated.

"I do not perceive that any particular advantage can accrue from giving specific formula of remedies in particular cases of insanity; for there are scarcely any two for which the same formula or dose would be suitable. Doses, like the remedies themselves, must be modified according to the constitution and peculiarities of the patient and symptoms of the case." (P. 580.)

It is important, says Dr. Burrows, to remark

"That cases of evident derangement of the intellectual faculties are sometimes met with, which, perhaps, on a very rigid examination, present no symptoms of corporeal disorder. All the functions seem regular, and there is no alteration in the external appearance, except, perhaps, a little more vivacity in the look, and a slight peculiarity in the eyes. These are persons in whom, invariably, the hereditary predisposition is inherent." (P. 581.)

If the word generally had stood in the place of "invariably," we should have offered no comment upon this passage. But two exceptions have fallen under our own observation to this absolute statement. In both, the patients have been insane for years. Not the slightest symptom of corporeal disorder has ever been manifested in either, with the exception of trifling rheumatic attacks from exposure to cold, in one lady. In neither of these persons was there any hereditary predisposition.

The various remedies that are commonly in use are now separately considered.

*General bloodletting* is in very general use. That it is much too inconsiderately and too indiscriminately employed, in this and many other cases, is a fact which, we believe, is daily attracting more observation. We trust, therefore, that the use of this powerful remedy will speedily be limited within more judicious bounds. "Many of eminent character among the moderns," says the author, "have doubted its efficacy; and experience has convinced me that, except in a very restricted sense, it is a practice fraught generally with mischief. Following example rather than experience, I tried depletion by bloodletting for several years; but, discovering my error, I became more cautious, and I believe that I have scarcely ordered venesection in six cases of simple mania or melancholia in as many years. My conclusion is, that, since I changed my practice, more have recovered; and certainly the cases have been less tedious and intractable." (P. 583.)

If any urgent necessity impels copious abstraction of blood in mania or melancholia, the more prudent practice, we are told, is to effect the object with the greatest celerity. Bleeding from the feet or ankles is not to be much relied upon. In the opinion of the author, it is only where a real state of plethora exists, or apoplexy is pending, that general bloodletting in mental derangement can be justified. The pulse at the radial artery is not a criterion we can depend upon.

The blood drawn in mania rarely exhibits the indications of inflammation. The results of anatomical investigation do not support the theory of cerebral inflammation in mania.

The sweeping condemnation both of the lancet and cupping in insanity, which has been passed by Dr. F. Willis, is deprecated. Dr. B. replies, that it is possible the one may be required; and he is sure that the other, or leeching, can seldom be dispensed with in any recent case. Bloodletting is never admissible in long-standing insanity, except a *temporary* attack has come on, with symptoms of active cerebral excitement.

*Topical bleeding* the author considers safe, if employed with moderate prudence. "In every case of recent insanity which I have seen, and I do not recollect an exception, local abstraction of blood from the head itself, or contiguous, as the nape of the neck or between the shoulders, has been indicated. The mode has been by cupping or by leeches. Cupping on the occiput is to be preferred."

There is much diversity of opinion as to the propriety of repeating local bleeding.

"Some conceive that the object is attained by a single emptying of the surcharged vessels of the brain; others repeat it through exacerbation and remission, even into the continuous form. The latter course I hold to be dangerous, as likely to produce a permanent state of collapse of the brain. My practice is to repeat cupping or leeches as long as symptoms of great cerebral excitation prevail, especially while a preternatural heat of the scalp is felt; but, when they remit, to desist from drawing away blood, and repeat it only with the renewal of these symptoms. If premonitory symptoms announce an attack, local depletion will often prevent it. Mere raving and fury must not be mistaken for cerebral excitation consequent on vascular excitement. They are probably the effect of that cerebral irritation which is produced by an opposite condition of the brain, and would inevitably be exasperated by any kind of depletion." (P. 591.)

Local determination occurs in the weak as well as the strong, and tonics and stimuli may be required to keep up the general tone, while topical bleeding may be useful. We may be allowed to add, that this fact, which is too commonly unattended to, should frequently lead to a similar principle of practice in many other diseases, especially palsy.

Dry cupping is occasionally useful, where even the smallest quantity of blood could not be detracted with safety.

"The blood is by this means derived from the surcharged internal vessels to those of the external, all of which will be seen greatly distended from the operation; and it is there retained awhile, without being absolutely withdrawn from the circulation, to the relief of the brain." (P. 594.)

*Refrigeration.*—The utility of this remedy, where there exists a preternatural heat of the head, is confessed in all cases of cerebral disorder.

"The natives of a part of India have two curious, and probably primitive, modes of lulling young children to sleep. The first is by the operation of a constant stream of cold water pouring on the crown of their heads. The parents wrap up the bodies and feet of the children warm in a blanket, and place them horizontally in trays; they then expose the vertex of the head only to the cooling influence of a running stream, and thus certainly induce repose."* (P. 597.)

The application of intense cold to the head in a chronic state of insanity, when the patient is noisy and violent, never induces quiescence and sleep. On the contrary, the brain being in a state of atony, cold then always becomes a source of irritation. The douche is not always safe, and requires to be used with precaution.

*Gyration and swinging.*—"In the intermitting form of insanity, gyration has been found of particular benefit in checking the approaching paroxysm. When a great prostration of strength suddenly succeeds to the full motion of the swing, most advantage is expected. Its effect in lowering the circulation and temperature of the body is so immediate, that alarm for the consequences is generally created in those not accustomed to the use of it.

"Where sleep is the wished-for object, a slow and long-continued action of the swing, if possible, without affecting the stomach to vomiting, is to be kept up.

"Like every other antimaniacal remedy yet prescribed, it is acknowledged that this sometimes entirely fails in producing the

* Frazer's Tour in the Himalaya Mountains, p. 105.

desired effect. Possibly, as it always occasions great apprehensions, its ordinary operations on the system are thereby counteracted.

"The operation of gyration, either vertically or horizontally, is strongly advised, as a moral as well as a medical agent in chronic cases; for, where no expectation of cure has been entertained, a few trials have produced a wonderful improvement in manners and behaviour.

"Where the degree of violence has been so great as to compel a rigid confinement, the patient has become tractable, and even kind and gentle, from its operation. The morbid association of ideas has been interrupted, and even the spell of the monomaniac's cherished delusion broken." (P. 602.)

*Sleep.*—The agents previously mentioned have all a tendency to induce sleep. It is here considered as a remedy in mental disorders. "Too much sleep (says our author,) disposes to all the disorders of a slow circulation, and to weakness and cachexy. In affections of the head generally, sleep does not alleviate; and it is possible that an abridgment of it in those afflicted with cerebral affections might even prove beneficial. However that may be, I am quite clear that there is commonly by far too great a solicitude to procure sleep in mental derangement." (P.605.) The medical attendant should remember, that if the mean used to employ sleep be not indicated by the physical state of the patient, mischief follows its exhibition.

"A maniac awoke from sleep artificially obtained, is a giant refreshed. New activity is imparted to the sensorium, and his muscular powers are recruited. If he have lost by it one hallucination, another assumes its place, more wild, perhaps, and extravagant than the former, and his waking dreams are the more vivid: hence his violence and raving are increased, and the power of continuing them prolonged." (P. 607.)

Sleep, when caused by any of the above-mentioned remedies, is desirable. The slumbers that result from these means are calm, and the excitement and activity of the brain are diminished. Plentiful ingestion increases the afflux of blood to the head, and where it is deficient, as in the cachectic, it operates as an anodyne. "Thus, a hearty meal for supper has been found to induce refreshing rest in maniacs, where every other means has failed. This perfectly accords with a well-known law in physiology, where the brain is in that condition which long-continued insanity produces." (P. 608.) Such an experiment must, of course, be very cautiously made.

*Narcotics.*—The most opposite opinions exist as to the exhibition of narcotics in insanity. Dr. Burrows probably accounts truly for these contradictions, by presuming that they arise chiefly from ignorance of the distinct stages which insanity assumes, or from not noting the exact state of the patient when the narcotic is given. Before opiates are administered, the system must be somewhat lowered, if the patient is of a full and strong habit.

"In the advanced stage of the disease, when, by local depletion of blood, vomiting, purging, and an antiphlogistic plan, the vascular excitement is moderated, or in cases of slight temporary delirium from some sympathetic affection, or those deliria which sometimes occur in weak and highly nervous people, from biliary derangements or some sudden moral affection, an adequate opiate will often at once remove the delirium. In delirium or mania from hepatic derangement, copious evacuation by the bowels ought to precede an opiate. In the delirium of simple nervous irritation and hysteric affections, provided there be no cerebral determination or congestion, (which, however, may always be suspected,) a sufficient opiate will carry it off. And should the blood-vessels of the head be evidently surcharged, they must be relieved before advantage can be expected from an anodyne." (P. 610.)

Weak opiates taken by the stomach are decidedly mischievous in mania. A large dose must be administered, attending to the above precautions, repeating smaller ones till the end be attained. To fix the maximum dose is impossible. Having mentioned the very large doses given by other physicians, the author observes,

"I have never ventured beyond five grains of purified opium as the first dose. In those cases where I have deemed an anodyne admissible, I generally begin with three grains, and repeat one every two or three hours. I have never in this way exceeded twelve grains; and if sleep has not then followed, I have desisted.' (P. 612.)

Battley's liquor opii sedativus affects the head less, nor does it constipate so much as laudanum. The strength of this preparation Dr. B. does not consider greater than that of the tincture of opium. We have formed the same opinion from our own experience; yet there are many who assert, with its inventor, that it is much more powerful.

Dupuytren has lately published some remarks upon the efficacy of small doses of laudanum given in clysters, not only in traumatic delirium, but in what he calls nervous delirium. We have at this moment a patient under our care who suffers severely from some obscure disease of the bladder and uterus. Large doses of opium taken by the mouth have proved less efficacious in relieving her torture than a suppository of two grains of solid opium introduced into the rectum.

*Hyoscyamus* is useful chiefly in those cases of nervous irritation which accompany great despondency, where it is necessary to obtain a state of quietude on which sleep may be expected to supervene. It neither stupefies nor constipates. Although we have used this medicine very frequently, the following remark has escaped us: "It is apt to produce a very dry mouth, and a blackish appearance of the tongue, which I have known raise alarm in those unacquainted with this effect of hyoscyamus."

Dr. Burrows has also tried, both in mania and melancholia, the extracts of stramonium, aconitum, and belladonna, as soporifics. "One grain of the former in furious mania has procured several hours' sleep, when other narcotics, in considerable doses, have not succeeded; but the patients, in all the cases, were infinitely more violent when they awoke. Nearly the same may be said of the two latter narcotics." (P. 618.) The same rule obtains in the use of all narcotics. They are not admissible during great vascular action or congestion of the brain, or a constipated state of the bowels.

The author makes no mention of the hemlock. We have found it useful in allaying very distressing restlessness of mind induced by great mental fatigue, when other remedies of the same class were not so beneficial.

*Blistering* "can never be serviceable in mania, any more than opium, where cerebral vascular excitement or congestion exists, till local or general depletion has preceded." If admissible at all, it is in the more advanced stages of insanity.

"But it is still preferable to excite vesication, where it is indicated in mania, by the application of a plaster composed of tartarised antimony and the common wax plaster. It soon occasions considerable heat and a crop of pustules, from which a discharge may be kept up or checked at will. This application might judiciously supersede cantharides in mania, since it produces all their good and none of their bad effects." (P. 621.)

*Setons and issues.*—The author has rarely met with a case where convalescence was the result of either of these applications. If retrocession of some cutaneous eruption has taken place, drains of these kinds may be useful. "Long established setons and issues hastily dried up have caused many cerebral affections, and insanity among them."

*Artificial eruptions.*—Tartarised antimony is the best agent for producing this effect. Dr. JENNER has spoken very highly of it.* Dr. Burrows has not met with the success he expected from this auxiliary. There are few cases in which he would venture to rub in this active preparation for weeks

* Letter to C. H. Parry, M.D. on the Influence of Artificial Eruptions.

together, as recommended by Dr. Jenner; "for it sometimes occasions very deep sloughs, which are not lightly to be treated in some cases of insanity."

Under the heads of bathing, purging, vomiting, nausea, salivation, digitalis, prussic acid, camphor, spirit of turpentine, tonics, tobacco, diet, and resistance of food, many good practical remarks are contained.

The fourth Commentary of this Part considers the moral treatment.

The volume concludes with a few hints upon medical evidence in cases of insanity.

The opinion we entertain of Dr. Burrows' book is expressed in the length of the analysis which we have given of it. We have rarely, indeed, had a more troublesome task; for every page is so pregnant with practical information, that we have had much difficulty in determining what parts we could pass over with propriety. Although we have devoted three articles to the consideration of the work, and have given, we hope, a more satisfactory sketch of its general merits than any of our contemporaries, we beg to impress upon our readers the absolute necessity of attentively studying the very masterly delineation which Dr. Burrows has given of that most important of subjects, mental derangement. Dr. B. has modestly observed, that he offers no novelty; he has merely collected facts. To this we reply, in defence of himself against his own concession, "*Si nihil dictum quod non dictum prius, methodus sola artificem ostendit.*"

