# Erratum in Our Last Number

**Published:** 1829-02

**Authors:** 


ERRATUM *in our last Number, p.* 69.—In the quotation from Dr. Burrows, p. 581, the last line but one, for "there are," read "these are." Without this alteration, the subsequent comment of the reviewer is scarcely intelligible.

